# An ultrasound-scanning *in vivo* light source

**DOI:** 10.21203/rs.3.rs-6773130/v1

**Published:** 2025-06-19

**Authors:** Shan Jiang, Marigold G. Malinao, Fan Yang, Yushun Zeng, Silky S. Hou, Xiang Wu, Nicholas J. Rommelfanger, Lata Chaunsali, Jun Ding, Xiaoke Chen, Qifa Zhou, Harald Sontheimer, Guosong Hong

**Affiliations:** 1 Department of Materials Science and Engineering, Stanford University, Stanford, CA, USA; 2 Wu Tsai Neurosciences Institute, Stanford University, Stanford, CA, USA; 3 Department of Neuroscience, University of Virginia, Charlottesville, VA, USA; 4 Alfred E. Mann Department of Biomedical Engineering, University of Southern California, Los Angeles, CA, USA; 5 Department of Applied Physics, Stanford University, Stanford, CA, USA; 6 Department of Neurosurgery, Stanford University School of Medicine, Stanford, CA, USA.; 7 Department of Neurology and Neurological Sciences, Stanford University, Stanford, CA, USA; 8 Department of Biology, Stanford University, Stanford, CA, USA.

## Abstract

Biological systems operate across distributed regions with fast, localized dynamics, yet existing biointerfaces fail short in providing both high spatiotemporal precision and the ability to dynamically target any region without disturbing surrounding tissue. Here, we present an *in vivo* deep-tissue light source based on focused ultrasound (FUS) scanning of mechanoluminescent nanotransducers (MLNTs) circulating through the vasculature. We demonstrate the programmability of this approach in tissue-mimicking phantoms and the endogenous circulatory system of animals, where tunable spatial resolution and dynamic light patterning can be achieved. We validate the functionality of the ultrasound-scanning light source in opsin-expressing neurons through electrophysiological recordings and immunostaining. We showcase dynamic three-dimensional brain targeting and temporally resolved behavioral control in freely moving animals via the ultrasound-scanning *in vivo* light source. This non-invasive deep-tissue light source offers a versatile strategy for body-wide optical interfacing.

## Introduction

Biological processes span anatomically distributed systems and unfold with rapid dynamics that are spatially localized^1–4^. Despite recent advances in technology interfacing with biological tissues, trade-offs between high spatiotemporal resolution and flexible spatial targeting remain a persistent constraint. Implantable devices can offer cellular-level precision but are inherently invasive and restricted to fixed locations^5–7^, limiting their utility for distributed or dynamic targeting. Conversely, while non-invasive approaches provide improved spatial flexibility compared to implantable devices^8,9^, most of them still face challenges, including limited penetration depth^10,11^, poor targeting ability in three dimensions, lack of cell-type specificity^12,13^, and slow response kinetics^14^.

Achieving dynamic targeting requires two complementary criteria: spatially flexible energy input and widespread presence of responsive agents whose effects rely on region-specific energy input. These conditions can be satisfied by pairing a repositionable, non-invasive energy source with a responsive agent that is broadly distributed throughout the body. In such designs, the energy source typically enables precise spatiotemporal control, while the agent ensures reliable biological responsiveness across extended tissue volume. Nonetheless, while many existing approaches leverage non-invasive energy sources to achieve biointerfacing, they typically require locally delivered responsive agents or implants^15–17^, limiting their system-wide scalability and applicability. For example, near-infrared (NIR) light offers moderate penetration, but often requires upconversion nanoparticles (UCNPs)^18^ or other transducing agents^19,20^ injected into specific regions to produce physiologically effective light or heat. Electromagnetic fields can stimulate deep tissues through magnetic nanoparticles^21–23^, yet these agents must also be strategically placed. Ultrasound, with its deep tissue penetration and high spatial resolution, has emerged as a powerful non-invasive modality^24,25^. Although existing ultrasound-based approaches have demonstrated the in situ generation of biologically effective signals such as light emission^26,27^, pharmaceutically active molecules^28^, or electrical activity^29^, it still remains a challenge to achieve body-wide biologically scanning with high spatiotemporal resolution.

**Fig. 1.**
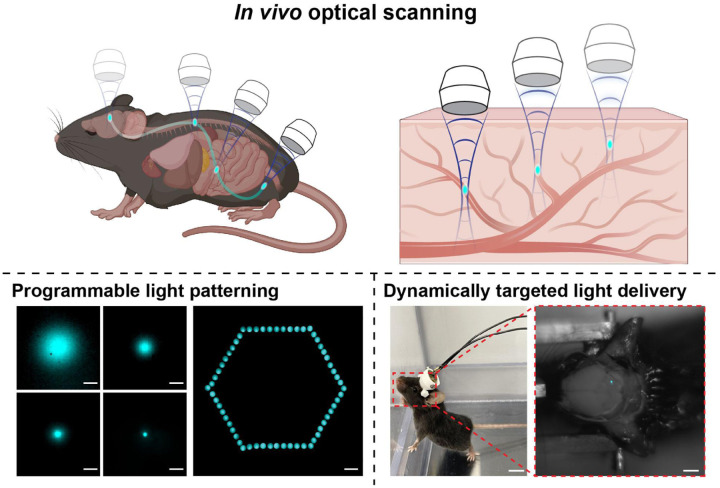
Optical scanning of biological systems via an ultrasound-mediated light source. The combination of relocatable FUS and the circulating mechanoluminescent nanotransducers (MLNTs) allows for dynamic optical scanning of the complex biological systems via the endogenous vascular network. This platform supports programmable light patterning (scale bars, 1 mm) and dynamically targeted light delivery (scale bars represent 1 cm and 5 mm, respectively).

In this work, we present an ultrasound-scanning *in vivo* light source that enables non-invasive, body-wide light delivery with high spatiotemporal resolution (Fig. 1). We first demonstrated spatially tunable focusing and light patterning in a tissue-mimicking phantom, showcasing raster-scanned light delivery with confocal-like targeting precision for programmable optical control. By leveraging focused ultrasound (FUS) to activate mechanoluminescent nanotransducers (MLNTs) circulating within the vasculature, our approach eliminates the need for implanted devices while supporting body-wide light emission encompassing multiple regions in the brain and peripheral organs. To demonstrate effective *in vivo* light delivery, we used animals with opsin-expressing neurons as illustrative models, validating light-induced responses through real-time electrophysiological recordings and postmortem immunostaining. We further showed that the ultrasound-scanning light source can dynamically target anatomically distinct brain regions, achieving spatially confined activation validated by immunostaining. Additionally, we applied the ultrasound-scanning light source to freely moving animals, demonstrating temporally and spatially resolved light-based modulation of behavior via targeted stimulation of genetically defined neuronal populations. Our approach establishes a non-invasive platform for whole-body interaction through deep-tissue light delivery, enabling programmable and precise control across space and time.

## Results

**Fig. 2.**
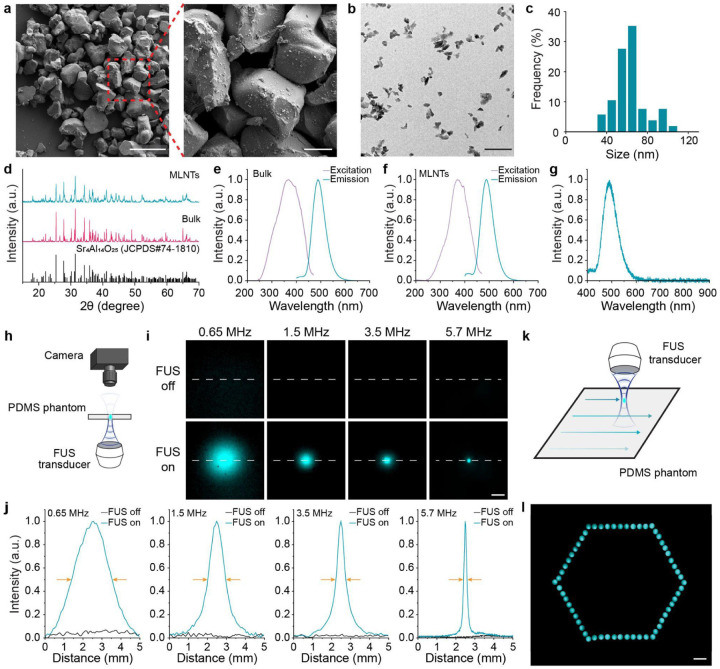
An ultrasound-scanning light source based on mechanoluminescent nanotransducers (MLNTs). **a**, SEM images of bulk mechanoluminescent materials. **b**, A TEM image of MLNTs. **c**, A histogram showing the size distribution of MLNTs. **d**, XRD spectra of bulk mechanoluminescent materials, MLNTs, and the JCPDS spectrum of Sr_4_Al_14_O_25_. **e-f**, Excitation (purple) and emission (cyan) spectra of bulk mechanoluminescent materials (**e**) and MLNTs (**f**). **g**, Mechanoluminescence spectrum of ultrasound-mediated light emission from an MLNT-doped PDMS phantom. **h**, A schematic illustration of imaging the ultrasound-mediated mechanoluminescence emission in a PDMS phantom. **i,** Representative mechanoluminescence images at the focus of the applied ultrasound in a PDMS phantom when ultrasound is off (top) and on (bottom) for incident ultrasound with different frequencies. **j**, Line intensity profiles corresponding to the white dashed lines in **i**. **k**, A schematic diagram showing dynamic ultrasound-induced optical patterning in a raster-scanning manner. **l**, A representative mechanoluminescence image exhibiting a hexagon pattern created by the scanning FUS. Scale bars represent 50 μm (**a**, left), 10 μm (**a**, right), 500 nm (**b**), and 1 mm (**i, l**), respectively.

To meet the demands of body-wide non-invasive light delivery, we developed an ultrasound-scanning light source based on circulation-deliverable MLNTs. Specifically, we synthesized biocompatible MLNTs composed of Sr_4_Al_14_O_25_:Eu,Dy^30,31^. Colloidal MLNTs were obtained from the bulk mechanoluminescent material, followed by PEGylation to yield stable dispersion in aqueous media^26^. As shown in the scanning electron microscopy (SEM) images in Fig. 2a, the bulk mechanoluminescent material presented an average size of above 10 μm, which was significantly reduced to 30–110 nm colloids as shown in the transmission electron microscopy (TEM) images of Fig. 2b. This small size distribution of colloidal Sr_4_Al_14_O_25_:Eu,Dy (Fig. 2c) enabled chronic stability of the MLNTs suspension for up to 1 week (**Supplementary Fig. 1**). Besides the improved colloidal stability, X-ray diffraction (XRD) analysis revealed that the diffraction pattern of MLNTs closely matched that of the bulk counterpart, confirming that the crystal structure of the host material remained unchanged in the colloidal MLNTs (Fig. 2d). After confirming the structural preservation, we next examined the optical properties of bulk and colloidal Sr_4_Al_14_O_25_:Eu,Dy materials, starting with the photoluminescence characteristics. The excitation and emission spectra of the bulk material (Fig. 2e) and MLNTs (Fig. 2f) exhibited identical spectral features, indicating the preservation of the optical properties of Sr_4_Al_14_O_25_:Eu,Dy in the colloidal form. Furthermore, the luminescence spectrum of MLNT-doped polydimethylsiloxane (PDMS) phantom under FUS reveals an identical emission peak at 490 nm (Fig. 2g), characteristic of typical mechanoluminescence behavior. A linear dependence of light emission intensity on incident ultrasound pressure confirms the origin of the emission as mechanoluminescence (**Supplementary Fig. 2**). The observed mechanoluminescence emission of Sr_4_Al_14_O_25_:Eu,Dy is believed to arise from an electric field-mediated trap activation process, triggered by grain-boundary dislocations moving during intergranular slipping under mechanical stress (**Supplementary Fig. 3**) despite the lack of piezoelectricity in the host material^30^.

We next characterized the achievable spatial resolution of light emission from MLNTs under FUS. Using ultrasound transducers with different central frequencies, we imaged the mechanoluminescence emission from a phantom doped with MLNTs (Fig. 2h). Specifically, ultrasound transducers with central frequencies of 0.65 MHz, 1.5 MHz, 3.5 MHz, and 5.7 MHz were tested. The resulting mechanoluminescence images (Fig. 2i) showed no detectable emission when the FUS was off, whereas FUS stimulation yielded distinct mechanoluminescence emission spots. Notably, the emission spot became progressively smaller at higher ultrasound frequencies, demonstrating that frequency tuning can acoustically shape the light emission, similar to lens-based focusing in optical systems. To further quantify the spatial distribution, line intensity profile analysis of mechanoluminescence images revealed the full width at half maximum (FWHM) of the emission spot as 2.1 mm, 0.94 mm, 0.52 mm, and 0.18 mm, respectively (Fig. 2i). In comparison, the theoretical FWHMs under diffraction-limited conditions were 1.6 mm, 0.70 mm, 0.30 mm, and 0.18 mm, respectively, in good agreement with the sizes of mechanoluminescence emission spots^32^.

Besides submillimeter spatial precision and tunable emission size, the relocatable nature of the ultrasound focus enables dynamic optical patterning in a raster-scanning manner (Fig. 2k). To demonstrate this capability, we sequentially produced 60 light emission spots forming a hexagonal shape by scanning the ultrasound focus while capturing the image of mechanoluminescence emission in a fixed field of view (Fig. 2l). This spatiotemporally controlled emission pattern underscores the potential of the ultrasound-scanning light source similar to a confocal microscopy for both raster scanning and precise optical targeting in three-dimensional organisms.

**Fig. 3.**
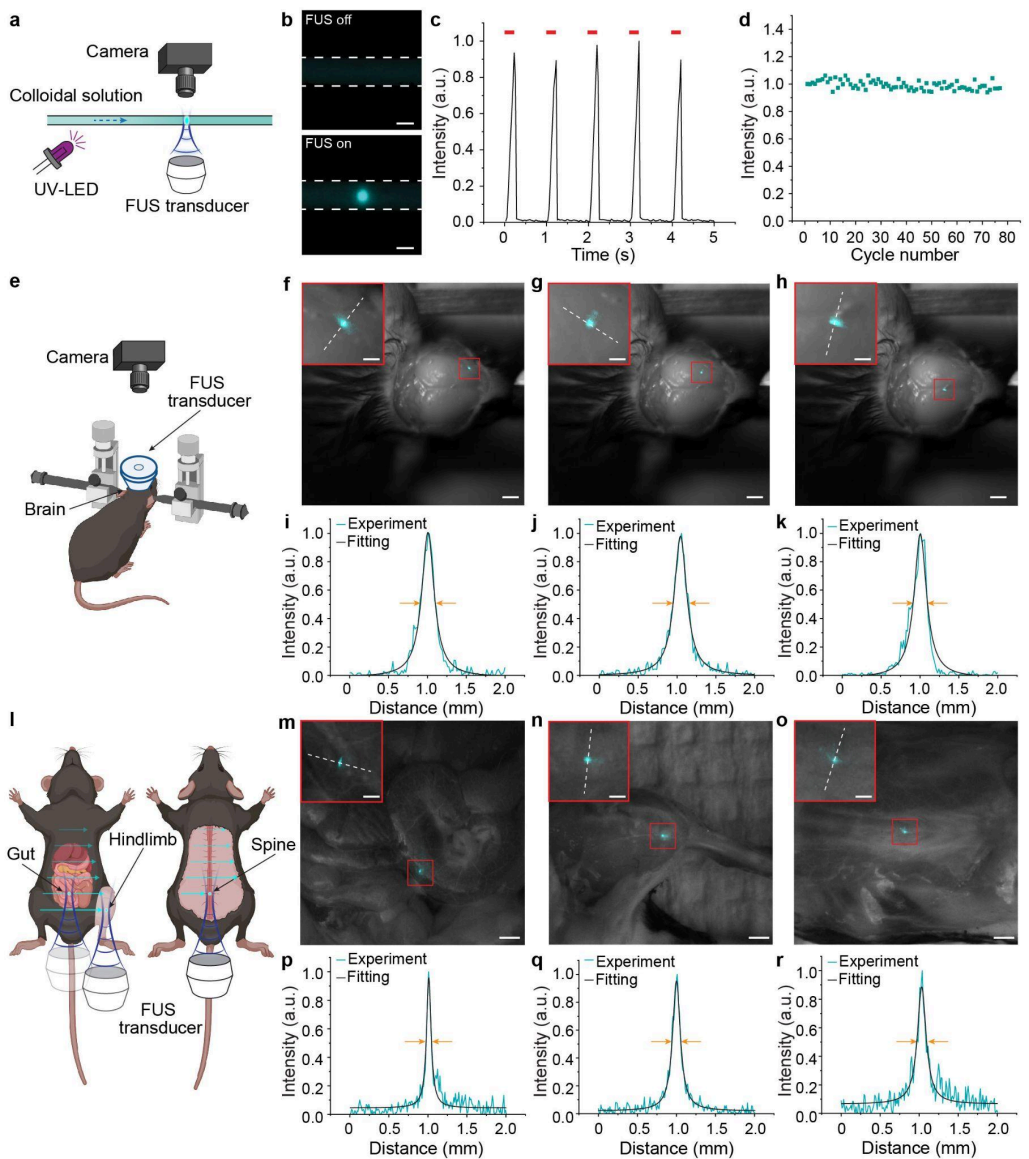
An ultrasound-scanning light source in circulatory systems. **a**, A schematic illustration showing the ultrasound-mediated light source enabled by an artificial circulatory system. **b**, Representative images of ultrasound-mediated mechanoluminescence emission from MLNTs circulating in the tubing, which is indicated by dashed lines, when FUS was off (top) and on (bottom). Scale bars represent 1 mm. **c**, Dynamics of light emission intensity from MLNTs in the artificial circulatory system, where the red ticks indicate the duration of FUS pulses. **d**, Normalized peak intensity of mechanoluminescence emission over many cycles. **e**, A schematic illustration showing the ultrasound-mediated light source enabled by MLNTs in cerebral vessels of the mouse brain. **f**-**h**, Overlay of brightfield images with FUS-induced emission from three representative regions: the primary motor cortex (M1, **f**), the primary somatosensory cortex (S1, **g**), and the retrosplenial agranular (RSA) cortex (**h**) with insets representing the mechanoluminescence emission from those corresponding regions indicated by the red box. Scale bars represent 2 mm in **f-h** and 500 μm in the insets. **i-k**, Line intensity profiles corresponding to the white dashed lines in the insets of **f-h**, respectively. **l**, A schematic illustration showing the ultrasound-mediated light source enabled by MLNTs in blood vessels in the gut, hindlimb, and spine by dynamically targeted FUS. **m-o**, Overlay of brightfield images with FUS-induced emission from the gut (**m**), the hindlimb (**n**), and the spine (**o**) with insets corresponding to regions indicated by the red box. Scale bars represent 2 mm in **m-o** and 500 μm in the insets. **p-r**, Line intensity profiles corresponding to the white dashed lines in the insets of the gut (**p**), limb (**q**), and spine (**r**), respectively.

The ability of FUS to dynamically generate light emission throughout a living organism relies on the delivery of MLNTs via the endogenous circulatory system. To demonstrate this feasibility, we first constructed an artificial circulatory system (Fig. 3a), in which MLNT solution flowed through a flexible tubing to recapitulate the vascular features in biological systems. The sharp contrast of MLNT solution when FUS was on and off (Fig. 3b) indicates the preserved mechanoluminescence properties under flow conditions. Moreover, the circulatory system maintained stable dynamic emission profiles (Fig. 3c), where the peak intensities remained constant over extended stimulation cycles (Fig. 3d). Our power density measurement of mechanoluminescence emission (**Supplementary Fig. 4**) and circulation dynamics of systemically delivered MLNTs (**Supplementary Fig. 5**) suggested sufficient optical power density of the FUS-mediated light source for a variety of light-based biological applications^33,34^ (see Supplementary Text).

Building on the results from the artificial circulatory system, we next evaluated the ultrasound-scanning light source in mice. Unlike the tubing with a uniform diameter in the artificial circulatory system, the intrinsic vascular network in animals is highly heterogeneous in a three-dimensional space^35^. This structural complexity affects the distribution of MLNTs and, along with the FUS beam profile, defines the spatial resolution of the ultrasound-scanning *in vivo* light source. To experimentally evaluate this spatial resolution under realistic biological conditions, we systemically delivered MLNT solution into the mouse bloodstream and applied FUS stimulation using a ring transducer with central openings that facilitate real-time visualization (Fig. 3e). We then assessed the scanning capability of the ultrasound-mediated light source by inducing spatially confined mechanoluminescence emission in three anatomically distinct cortical regions: the primary motor cortex (M1, Fig. 3f), the primary somatosensory cortex (S1, Fig. 3g), and the retrosplenial agranular (RSA) cortex (Fig. 3h). The FWHM of the light emission spots was measured to be approximately 200 μm in the three targeted cortical regions (Fig. 3i–k). While mechanoluminescence emission was only captured from shallow cortical regions, the use of tissue-penetrant FUS could, in principle, generate light in deeper brain structures; however, this emission may not be detectable due to scattering within the overlying tissue^36^.

In addition to light emission in the brain, the ultrasound-scanning light source also enabled body-wide light delivery, such as in the gut, the hindlimb, and the spine (Fig. 3l). Light emission was successfully observed in these regions under FUS stimulation (Fig. 3m-o and **Supplementary Fig. 6**). Consistent with the light emission in cortical brain regions, the emission spots exhibited tight spatial localization with FWHM in the 100–200 μm range in these three organs (Fig. 3p-r). In some cases, we found that the ultrasound focus was sufficiently large to encompass multiple blood vessels, as evidenced by the illumination of several vessels in the mechanoluminescence images (**Supplementary Fig. 7**). This scenario is particularly advantageous, as cells located between vessels can receive light emitted from surrounding vessels in all directions, ensuring effective biomodulation by locally generated light (Supplementary Text).

**Fig. 4.**
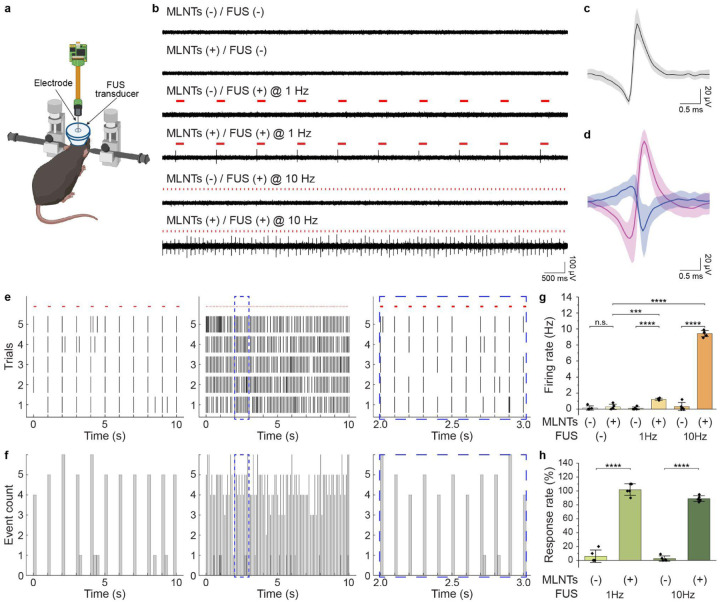
Validating the efficacy of the ultrasound-scanning *in vivo* light source with electrophysiological recording. **a**, A schematic illustration showing electrophysiological recording with simultaneous FUS-mediated light delivery in the live mouse brain. **b**, Representative electrophysiological recording traces under different experimental conditions. **c-d**, Overlay of sorted spikes, which represent putative neurons recorded at FUS modulation frequency of 1 Hz (**c**) and 10 Hz (**d**) on two different electrodes. The L-ratio and isolation distance for the two clusters shown in **d** are 0.0014 and 60.01, respectively. In each graph, the solid line represents the averaged spike waveform, while the shaded area represents the standard deviation. **e**, Raster plots for five 10-s recording trials under the FUS-mediated light source modulated at 1 Hz (left) and 10 Hz (middle). The raster plot on the right presents a zoomed-in view of the 2–3 s time window (blue dashed box) in the middle plot. Each vertical tick represents a single-neuron firing event. Red horizontal ticks represent the duration of FUS pulses. **f**, Activity histograms of **e**, where the bin sizes for 1 Hz (left) and 10 Hz (middle) light sources are 200 ms and 20 ms, respectively. The right panel presents a zoomed-in view of the 2–3 s time window (blue dashed box) in the middle plot. **g**, Statistical analysis of firing rates of putative single units under different experimental conditions. One-way ANOVA, FUS(−): MLNTs(−) vs MLNTs(+), *F*_(1, 8)_=0.56, *P*=0.48; 1 Hz: *F*_(1, 8)_=133.45, *P*<0.0001; 10 Hz: *F*_(1, 8_)=1024.88, *P*<0.0001; MLNTs(+): FUS(−) vs FUS(+) @ 1 Hz, *F*_(1, 8)_=35.63, *P*=0.00033; MLNTs(+): FUS(−) vs FUS(+) @ 10 Hz, *F*_(1, 8)_=1644.48, *P*<0.0001. **h**, Statistical analysis of neuronal response rates under different conditions. One-way ANOVA, 1 Hz: *F*_(1, 8)_=307.2, *P*<0.0001; 10 Hz: *F*_(1, 8)_=1173.74, *P*<0.0001. Data shown in **g&h** are presented as mean ± s.d. and each point indicates an independent trial from n=5 in each group. *P* ≥ 0.05 (n.s.), ***P<0.001, ****P<0.0001.

We then sought to demonstrate the utility of the ultrasound-scanning light source for stimulating opsin-expressing neurons in live mice, using *in vivo* electrophysiological recording as the readout. As a representative example, we employed transgenic mice expressing the light-sensitive ion channel, Channelrhodopsin-2 (ChR2), under the Thy1 promoter (i.e., Thy1-ChR2 mice; Fig. 4a). Flexible fiber-based neural probes were used for simultaneous electrophysiological recording to minimize ultrasound-induced mechanical artifacts^37^. To validate the efficacy of FUS-mediated light emission, we designed six experimental conditions by varying the presence of MLNTs (MLNT+ or MLNT−) and the pulse repetition rate of FUS stimulation (none, 1 Hz, or 10 Hz) in the same Thy1-ChR2 mice. As shown in Fig. 4b, single-unit spiking activity was only observed when MLNTs were administered and FUS stimulation was applied (either at 1 Hz or 10 Hz), confirming the sufficiency and necessity of both MLNTs and FUS to induce robust ultrasound-mediated optogenetic stimulation of neurons. Importantly, the lack of responses to FUS in the absence of MLNTs rules out the alternative activation mechanism of neuron activation via ultrasound-induced stimulation of mechanosensitive ion channels^38^. Raw and bandpass-filtered traces confirm that the recorded spiking activity originated from neuronal responses and was not attributable to mechanical artifacts induced by FUS (**Supplementary Fig. 8**). We next performed principal component analysis (PCA) to isolate putative single-unit activity from these recordings. Average waveforms of representative units (Fig. 4c&d) and the entrainment of these spikes (Fig. 4e–g) to ultrasound pulses confirm successful activation of opsin-expressing neurons with the ultrasound-mediated light source. Specifically, a response rate near 100% was observed in these recorded neurons only when MLNTs were circulating in the bloodstream (Fig. 4h), confirming that the neuronal responses resulted from ultrasound-mediated light activation of opsins, rather than nonspecific ultrasound effects on neurons^39,40^.

**Fig. 5.**
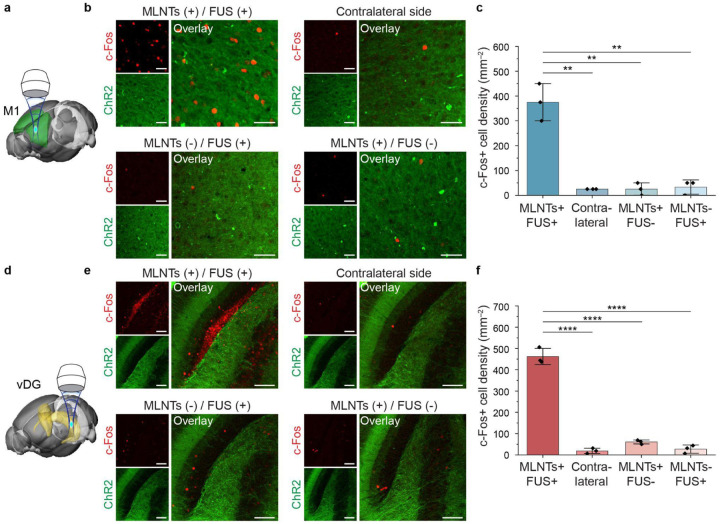
Validating the efficacy of the ultrasound-scanning *in vivo* light source with c-Fos immunostaining. **a**, Schematic illustration of the FUS-mediated light source in the M1 region. **b**, Representative immunostaining images of ChR2 and c-Fos in the M1 region under different experimental conditions. **c**, Statistical analysis of the c-Fos cell density in the M1 region across different experimental groups. One-way ANOVA, MLNTs(+)/FUS(+) vs contralateral side: *F*_(1,4)_=65.33, *P=*0.0013; MLNTs(+)/FUS(+) vs MLNTs(+)/FUS(−): *F*_(1,4)_=58.8, *P=*0.0016; MLNTs(+)/FUS(+) vs MLNTs(−)/FUS(+): *F*_(1,4)_=54.23, *P=*0.0018. **d,** Schematic illustration of the FUS-mediated light source in the ventral dentate gyrus (vDG). **e,** Representative immunostaining images of ChR2 and c-Fos in the vDG under different experimental conditions. **f,** Statistical analysis of the c-Fos cell density in the vDG across different experimental groups. One-way ANOVA, MLNTs(+)/FUS(+) vs contralateral side: *F*_(1,4)_=386.85, *P*<0.0001; MLNTs(+)/FUS(+) vs MLNTs(+)/FUS(−): *F*_(1,4)_=315.67, *P*<0.0001; MLNTs(+)/FUS(+) vs MLNTs(−)/FUS(+): *F*_(1,4)_=314.25, *P*<0.0001. Scale bars represent 40 μm in **b** and 80 μm in **e**. All data are presented as mean ± s.d. with data points shown for n = 3 mice in each group. **P < 0.01, ****P<0.0001.

Besides electrophysiological recordings, we next used c-Fos immunostaining to validate neuron responses to ultrasound-mediated light emission^41^. We first targeted the M1 region (Fig. 5a) and compared c-Fos expression levels between the experimental group (i.e., MLNT+/FUS+) and three control groups, including an internal control (i.e., the contralateral M1 region of the same animal), FUS stimulation without MLNTs (i.e., MLNT−/FUS+), and MLNTs injection without FUS stimulation (i.e., MLNT+/FUS−). Albeit shallow, the M1 region cannot be directly activated with transcranial illumination due to strong attenuation of the skull (**Supplementary Fig. 9**)^42^. In contrast, immunostaining of c-Fos in the M1 region under FUS-mediated light emission shows elevated c-Fos expression when both MLNTs and FUS were present (Fig. 5b&c), confirming the ability of ultrasound to penetrate the skull to produce localized light emission in the brain. The lack of c-Fos+ cells in control groups further confirms the sufficiency and necessity of both MLNTs and FUS in activating ChR2-expressing neurons. Additionally, c-Fos expression revealed an activation volume similar to that of the ultrasound focus, with a lateral FWHM of 341 μm, an axial FWHM of 1.4 mm, and a total activation volume of 0.085 mm^3^ (**Supplementary Fig. 10**). This consistent activation volume validates that light emission, after being produced in the blood, can easily cross the blood-brain barrier (BBB) and penetrate the adjacent brain parenchyma to activate neurons, without requiring the MLNTs themselves to traverse the BBB.

**Fig. 6.**
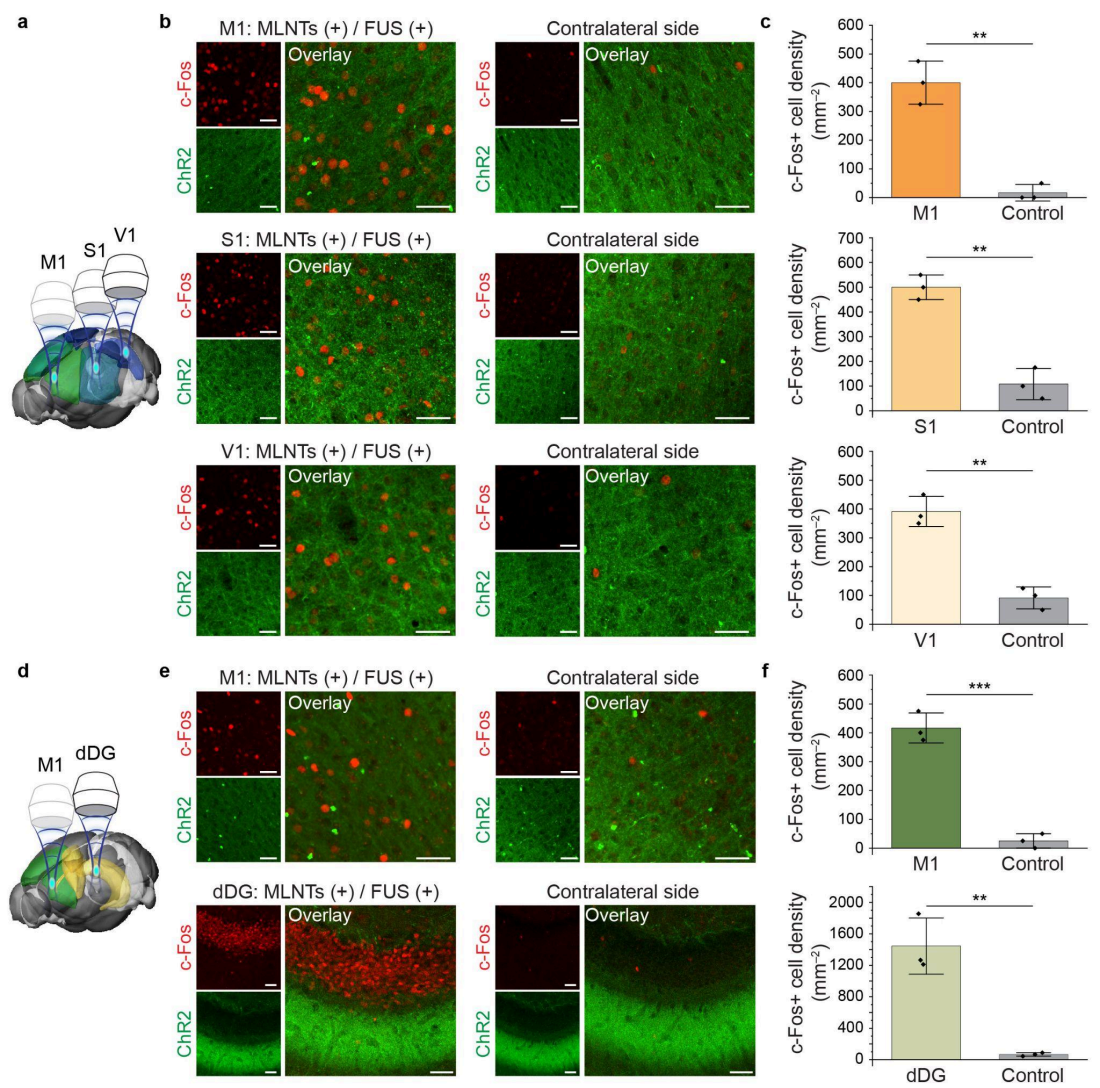
Validating the efficacy of the ultrasound-scanning light source that dynamically targets multiple brain regions. **a**, Schematic illustration of the ultrasound-scanning light source in three brain regions, the M1, the S1, and the primary visual cortex (V1) in the same animal. **b**, Representative immunostaining images of ChR2 and c-Fos in the M1, S1, and V1 regions after being stimulated by the ultrasound-scanning light source along with their corresponding regions in the contralateral hemisphere. **c**, Statistical analysis of the c-Fos cell density from ipsilateral and contralateral regions in **b**. One-way ANOVA, M1 (top): *F*_(1, 4)_=68.26, *P*=0.0012; S1 (middle): *F*_(1, 4)_=71.26, *P*=0.0011; V1 (bottom): *F*_(1, 4)_=64.8, *P*=0.0013. **d**, Schematic illustration of the ultrasound-scanning light source in two brain regions, the M1 region and the dorsal dentate gyrus (dDG) region. **e**, Representative immunostaining images of ChR2 and c-Fos in the M1 and dDG regions after being stimulated by the ultrasound-scanning light source and their corresponding regions in the contralateral hemisphere. **f**, Statistical analysis of the c-Fos cell density from ipsilateral and contralateral regions in **e**. One-way ANOVA, M1 (top): *F*_(1,_ 4)=138.06, *P*=0.0003; dDG (bottom): *F*_(1, 4)_=44.48, *P*=0.0026. All scale bars represent 40 μm. All data are presented as mean ± s.d. with data points shown for n = 3 mice in each group. **P < 0.01, ***P<0.001.

As demonstrated above, one salient advantage of the ultrasound-scanning light source is the capability of delivering light to tissues at various locations. To this end, we leveraged the dynamic targeting ability of the FUS-mediated light source to activate neurons across distinct regions within the same animal. First, by targeting M1, S1, the primary visual cortex (V1), and the ventral dentate gyrus (vDG) separately, we achieved successful activation in each of these regions with minimal crosstalks in other non-targeted regions, as confirmed by c-Fos immunostaining (**Supplementary Fig. 11**, Fig. 5d–f). Next, by sequentially relocating the ultrasound-scanning light source to M1, S1, and V1 of the same animal, we achieved activation of all three regions in the ipsilateral side receiving FUS, in contrast to contralateral controls, demonstrating programmable light emission across multiple cortical regions (Fig. 6a–c). Leveraging the volumetric targeting ability of FUS, we produced light emission both in a cortical region of M1 and a subcortical region of dorsal dentate gyrus (dDG) in the same animal, resulting in region-specific c-Fos activation in anatomically distinct targets *in vivo* (Fig. 6d–f).

**Fig. 7.**
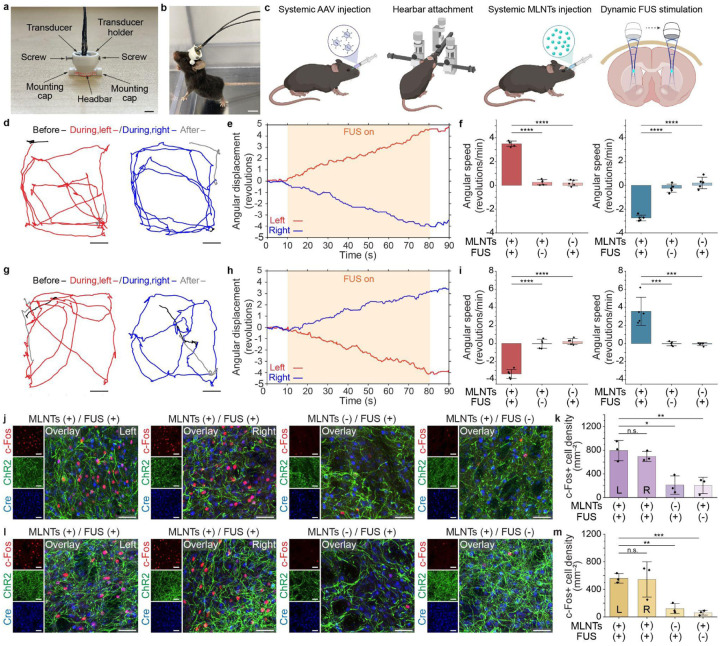
Ultrasound-scanning light source enables dynamic targeting of distinct deep-brain regions in free-moving mice. **a**, A photograph of the assembled head-mounting system. The scale bar represents 5 mm. **b**, A photograph of a mouse tethered to the wearable transducer. The scale bar represents 1 cm. **c**, Schematic illustration of the experimental procedure for systemic AAV and MLNT delivery and dynamic targeting of distinct deep-brain regions with FUS. **d**, **g**, Representative trajectories of a D1-Cre::ChR2-YFP mouse (**d**) and an A2a-Cre::ChR2-YFP mouse (**g**) before, during, and after stimulation by the ultrasound-scanning light source in the left (left) and right striatum (right). Scale bars represent 5 cm. The trajectories before, during, and after stimulation are color-coded in black, red (light in left striatum) or blue (light in right striatum), and grey, respectively. The trajectories were derived from video recordings captured from the bottom of the cage, presenting an inverted representation of the actual rotational direction. **e**, **h**, Angular displacement in revolutions of D1-Cre::ChR2-YFP mouse (**e**) and A2a-Cre::ChR2-YFP mouse (**h**) before, during, and after stimulation by the ultrasound-scanning light source, with red and blue lines indicating the focus of ultrasound in the left and right striatum, respectively. Positive values indicate counter-clockwise revolutions, while negative values indicate clockwise revolutions. The orange shades indicate the periods during which FUS stimulation was applied. **f**, **i**, Statistical analysis of rotational movements in terms of angular speed of D1-Cre::ChR2-YFP mice (**f**) and A2a-Cre::ChR2-YFP mice (**i**) with the ultrasound focus in the left striatum (left, red) and the right striatum (right, blue) under different experimental conditions. One-way ANOVA, MLNTs(+)/FUS(+) vs MLNTs(+)/FUS(−): D1 (left), *F*_(1, 8_)=515.85, *P*<0.0001; D1 (right), *F*_(1, 8)_=180.68, *P*<0.0001; A2a (left), *F*_(1, 8)_=112.25, *P*<0.0001; A2a (right), *F*_(1, 8)_=25.76, *P=*0.00096. MLNTs(+)/FUS(+) vs MLNTs(−)/FUS(+): D1 (left), *F*_(1, 8_)=469.33, *P*<0.0001; D1 (right), *F*_(1, 8)_=526.55, *P*<0.0001; A2a (left), *F*_(1, 8)_=131.19, *P*<0.0001; A2a (right), *F*_(1, 8)_=25.49, *P=*0.00099. **j, l**, Representative immunostaining images of ChR2, Cre, and c-Fos in the striatum region of D1-Cre::ChR2-YFP mice (**j**) and A2a-Cre::ChR2-YFP mice (**l**) under different experimental conditions. Scale bars represent 40 μm. **k**, **m**, Statistical analysis of c-Fos cell density of striatum region from D1-Cre::ChR2-YFP mice (**k**) and A2a-Cre::ChR2-YFP mice (**m**). One-way ANOVA, MLNTs(+)/FUS(+) left hemisphere vs right hemisphere: D1, *F*_(1, 4)_=0.79, *P=*0.42; A2a, *F*_(1, 4)_=0.010, *P=*0.93. MLNTs(+)/FUS(+) left hemisphere vs MLNTs(−)/FUS(+): D1, *F*_(1, 4)_=19.13, *P=*0.012; A2a, *F*_(1, 4)_=57.02, *P=*0.0017. MLNTs(+)/FUS(+) left hemisphere vs MLNTs(+)/FUS(−): D1, *F*_(1, 4)_=21.57, *P=*0.0097; A2a, *F*_(1, 4_)=124.46, *P=*0.00037. All data are presented as mean ± s.d. with data points shown for each animal from n = 5 mice in each group for the behavior study and n = 3 mice in each group for the immunohistology study. *P* ≥ 0.05 (n.s.), *P < 0.05, **P<0.01, ***P < 0.001, ****P<0.0001.

Having confirmed the efficacy of the ultrasound-scanning light source with electrophysiology and immunostaining, we next demonstrated its utility in freely moving animals. To implement dynamic light delivery in this setting, we designed a lightweight (0.05 g for chronic attachment; 0.78 g during behavioral experiment), head-mounting system (**Supplementary Fig. 12**) that provides compact housing of a home-made ultrasound transducer (see methods and **Supplementary Fig. 13**). A key advantage of this system (Fig. 7a) is its ability to allow easy, real-time adjustment of the ultrasound focus in the brain of freely-moving mice. Chronic stability of the system is validated by the normal ambulatory function of mice despite the headbar attached on their head for three months (Fig. 7b). We also validated that the head-mounting system enabled precise repositioning of the FUS transducer to spatially modulate light emission (**Supplementary Fig. 14**). In addition, connecting the wearable transducer to a commutator did not affect mechanoluminescence intensity (**Supplementary Fig. 15**). Mean squared displacement (MSD) analysis^43^ in freely moving mice demonstrates that the head-mounting system did not interfere with natural locomotion (**Supplementary Fig. 16**).

We next demonstrated the utility of the ultrasound-scanning light source in modulating the ambulatory behavior of mice via the compact headstage. We selected two functionally distinct pathways within the basal ganglia, composed of D1-expressing (direct) and A2a-expressing (indirect) projection neurons located in the striatum, a subcortical structure critical for motor planning and action selection^44,45^. Specifically, D1-Cre and A2a-Cre mice received systemic injections of a Cre-dependent ChR2-expressing adeno-associated virus (AAV-PHP.eB-EF1a-double-floxed-hChR2(H134R)-EYFP), which enables minimally invasive, global gene transduction across the blood-brain barrier for cell-type-specific expression (Fig. 7c)^46,47^. Following viral transduction, a headbar was attached to the resulting D1-Cre::ChR2-YFP and A2a-Cre::ChR2-YFP mice (**Supplementary Fig. 17**)^48^, facilitating subsequent dynamically targeted light emission via the wearable transducer.

Behavioral experiments in D1-Cre::ChR2-YFP mice demonstrated that the ultrasound-scanning *in vivo* light source effectively induced dynamically-adjusted directional movement. Specifically, light emission produced in the left or right striatum of the same animal elicited consistent circling behavior in opposite directions, confirming region-specific neuromodulation with this dynamic light source (Supplementary Movies 1–2, Fig. 7d–e). This directional circling behavior was further supported by a significant increase in rotational speed, as shown in Fig. 7f and **Supplementary Fig. 18**, only observable when light was produced at the ultrasound focus in the MLNTs+/FUS+/ChR2+ group. In contrast, A2a-Cre::ChR2-YFP mice exhibited opposite turning behavior under the same stimulation paradigm (Fig. 7g–i, **Supplementary Fig. 18**, Supplementary Movies 3–4), consistent with the opposite functional roles of the indirect and direct pathways in the basal ganglia^49,50^. This behavioral experiment demonstrates the unique ability of the dynamically targeted *in vivo* light source to engage distinct neural circuits at different regions in the same brain.

To further confirm that the observed behavioral changes were driven by the neuron-specific activation via the dynamically targeted light source, we performed c-Fos immunostaining of D1-Cre::ChR2-YFP and A2a-Cre::ChR2-YFP mice after receiving the stimulation in the striatum of both hemispheres. As shown in Fig. 7j&l, robust c-Fos expression was observed in both hemispheres, with no significant difference between the left and right sides (Fig. 7k&m). The absence of hemispheric differences in c-Fos expression demonstrates that our head-mounting system can reliably shift the light emission spot between brain regions to consistently deliver sufficient photons for activating ChR2-expressing neurons. Besides, the c-Fos levels in the MLNTs+/FUS+/ChR2+ group were significantly higher than in any of the control groups lacking MLNTs, FUS, or ChR2 expression, as shown in Fig. 7k&m and **Supplementary Fig. 19**. Together, these findings confirm that the rotational behaviors observed in freely moving animals were driven by region-specific light emission via the ultrasound-scanning light source.

We next conducted a comprehensive biocompatibility study to evaluate the long-term systemic effects of the ultrasound-scanning light source. Specifically, biodistribution analysis and pharmacokinetic studies confirmed gradual excretion of MLNTs through urine and feces over the course of 1 week (**Supplementary Figs. 5, 20, and 21**). Complete blood count (CBC) tests at 1 week and 4 weeks post-injection indicated minimal systemic toxicity (**Supplementary Fig. 22**). While most hematological parameters remained within normal ranges, a modest increase in white blood cell count was observed in mice injected with MLNTs, warranting further investigation. Complementary histological evaluation using hematoxylin and eosin (H&E) staining at the same time points revealed no inflammation, necrosis, or structural abnormalities in major organs, confirming preserved tissue integrity at the morphological level (**Supplementary Fig. 23**).

**Fig. 8.**
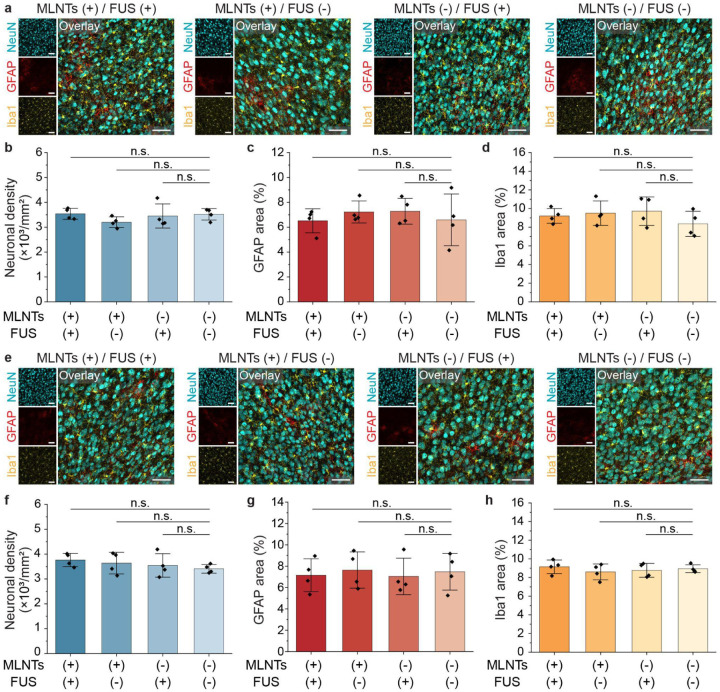
Biocompatibility assessment of the ultrasound-scanning light source. **a**, **e**, Representative immunostaining images of the M1 region 1 week (**a**) and 4 weeks (**e**) post-procedure under different experimental conditions. **b**, **f**, Statistical analysis of neuronal density 1 week (**b**) and 4 weeks (**f**) post-procedure. One-way ANOVA, MLNTs(+)/FUS(+) vs MLNTs(−)/FUS(−): 1 week, *F*_(1, 6)_=0.02, *P*=0.89; 4 weeks, *F*_(1, 6)_=5.04, *P*=0.07. MLNTs(+)/FUS(−) vs MLNTs(−)/FUS(−): 1 week, *F*_(1, 6)_=3.93, *P*=0.94; 4 weeks, *F*_(1, 6)_=0.92, *P=*0.37. MLNTs(−)/FUS(+) vs MLNTs(−)/FUS(−): 1 week, *F*_(1, 6)_=0.06, *P*=0.81; 4 weeks, *F*_(1, 6)_=0.27, *P*=0.62. **c**, **g**, Statistical analysis of GFAP area 1 week (**c**) and 4 weeks (**g**) post-procedure. One-way ANOVA, MLNTs(+)/FUS(+) vs MLNTs(−)/FUS(−): 1 week, *F*_(1, 6)_=0.00, *P*=0.95; 4 weeks, *F*_(1, 6)_=0.08, *P*=0.79. MLNTs(+)/FUS(−) vs MLNTs(−)/FUS(−): 1 week, *F*_(1, 6)_=0.31, *P*=0.60; 4 weeks, *F*_(1, 6)_=0.02, *P*=0.89. MLNTs(−)/FUS(+) vs MLNTs(−)/FUS(−): 1 week, *F*_(1, 6)_=0.35, *P*=0.57; 4 weeks, *F*_(1, 6)_=0.13, *P*=0.73. **d**, **h**, Statistical analysis of Iba1 area 1 week (**d**) and 4 weeks (**h**) post-procedure. One-way ANOVA, MLNTs(+)/FUS(+) vs MLNTs(−)/FUS(−): 1 week, *F*_(1, 6)_=1.19, *P*=0.32; 4 weeks, *F*_(1, 6)_=0.24, *P*=0.64. MLNTs(+)/FUS(−) vs MLNTs(−)/FUS(−): 1 week, *F*_(1, 6)_=1.50, *P*=0.27; 4 weeks, *F*_(1, 6)_=0.50, *P*=0.51. MLNTs(−)/FUS(+) vs MLNTs(−)/FUS(−): 1 week, *F*_(1, 6)_=1.77, *P*=0.23; 4 weeks, *F*_(1, 6)_=0.16, *P*=0.70. All scale bars represent 50 μm. All data are presented as mean ± s.d. with data points shown for each animal from n = 4 mice in each group. *P* ≥ 0.05 (n.s.).

Lastly, we performed immunostaining assays to evaluate the impact of the ultrasound-mediated light source on the neural tissue at 1 week and 4 weeks after the generation of light emission under FUS (Fig. 8). Four experimental groups were included: MLNTs+/FUS+, MLNTs+/FUS−, MLNTs−/FUS+, and MLNTs−/FUS− (negative control). Quantitative analysis of neuronal density (NeuN), astrocytic reactivity (GFAP), and microglial activation (Iba1) revealed no significant changes in any group compared to the negative control (MLNTs−/FUS−), indicating no observable neuroinflammation or difference in neuron density induced by the ultrasound-mediated *in vivo* light source. Collectively, we have established an ultrasound-scanning *in vivo* light source that enables dynamically adjustable light delivery with high spatiotemporal precision across the body, which surpasses the capabilities of existing approaches in achieving deep, targeted, and dynamic control of biological activity.

## Discussion

We report an ultrasound-scanning *in vivo* light source that enables programmable, non-invasive light delivery throughout the whole body using systemically circulating MLNTs and FUS. We experimentally validated this body-wide optical access by capturing focused light emission in multiple distinct regions, including various cortical areas of the brain, and peripheral organs such as the gut, the hindlimb, and the spine, with submillimeter spatial resolution. The temporal precision of this light source was confirmed by simultaneous *in vivo* electrophysiological recordings, where time-locked, single-unit neural responses were reliably evoked by the FUS-mediated light emission at various pulse repetition rates. Spatial flexibility was further showcased by dynamically targeting multiple cortical and subcortical regions in the same animal by stereotaxic repositioning of the focus of ultrasound, enabling region-specific optical control of neuron activity confirmed by immunostaining. This platform was also successfully applied in freely moving animals, achieving dynamic, non-invasive, real-time photostimulation of genetically defined striatal neurons with behavioral readouts.

There exist several confounding effects when ultrasound is applied to biological tissues. First, the FDA-suggested mechanical index threshold for cavitation (1.9 MPa/MHz^1/2^)^51^ yields pressure values of 2.33 MPa and 4.55 MPa corresponding to frequencies of 1.5 MHz and 5.7 MHz used in this study. Both values are far greater than that required for producing light emission (i.e., 1 MPa). Second, although ultrasound has been reported to cause nonspecific neuromodulatory effects, such effects are typically associated with low-frequency and high pulse repetition rate paradigms^52,53^, whereas our stimulation employs high frequencies and low pulse repetition rates. In addition, control experiments using FUS alone showed no neuromodulatory effects, as confirmed by electrophysiological recordings (Fig. 4, **Supplementary Fig. 8**), immunostaining (Fig. 5, **Supplementary Fig. 19**), and behavioral analysis (Fig. 7, **Supplementary Fig. 18**), further ruling out ultrasound-induced nonspecific activation. Lastly, to rule out potential thermal contributions, we monitored brain temperature during continuous FUS stimulation which revealed a negligible increase (<0.2 °C over 1 min; **Supplementary Fig. 24**) unlikely to interfere with neuronal activity^54^. These findings collectively support the use of our FUS-scanning light source as a non-invasive platform for deep-tissue photostimulation without confounding effects from ultrasound.

Although the present study used functional neuromodulation in the brain to demonstrate the utility of the ultrasound-mediated light source, our imaging results further confirmed that light emission can also be achieved in peripheral organs such as the gut, limb, and spine (Fig. 3, **Supplementary Figs. 6–7**). These findings demonstrate that the FUS-scanning light source enables light emission in any vascularized tissue, with spatial localization shaped by the interplay between the acoustic beam profile and local vessel distribution. Such versatility in spatiotemporally dynamic light delivery supports a broad spectrum of light-based interventions, such as gene editing^55,56^, photodynamic therapy^57,58^, deep-tissue imaging^59,60^, and light-triggered drug release^61,62^. These capabilities highlight the potential of this system as a generalizable, non-invasive platform for programmable photomodulation across organ systems and biological functions beyond the nervous system.

Furthermore, the spatial resolution of the ultrasound-mediated light delivery can be enhanced through advanced transducer designs, including crossed-beam phased arrays^63,64^. Besides, by incorporating multi-element transducers, it may be possible to transition from sequential to simultaneous multi-region light delivery, expanding the potential for real-time, parallelized modulation spanning complex biological systems. While our current head-mounting setup serves as a lightweight prototype, it may be further advanced by integrating a motorized 3D linear stage or robotic positioning module, enabling automated and rapid transducer repositioning for large-scale, volumetric light delivery.

From an engineering perspective, the ultrasound-scanning *in vivo* light source introduces a scalable and modular platform for precise, non-invasive control of internal biological targets with light. Unlike existing modalities limited by fixed implants, shallow penetration, or poor temporal resolution, this system achieves dynamic, volumetric light delivery through the unique pairing of circulating mechanoluminescent agents and repositionable ultrasound input. Its compatibility with non-invasive stimulation, multi-region access, and integration with head-mounting systems opens an ideal platform for biointerfacing covering the whole body. As such, this approach lays the foundation for a new class of distributed, light-based biotechnologies with applications across physiology, neurobiology, medicine, imaging, and beyond.

## Methods

### Synthesis of mechanoluminescent nanotransducers (MLNTs).

The bulk mechanoluminescent materials of Sr_4_Al_14_O_25_:Eu,Dy (SKU:P02-AQU-F008Z; TechnoGlow, Ennis, TX) was ball-milled (SPEX SamplePrep, Metuchen, NJ) with oleic acid (CAS#112-80-1, Fisher Scientific, Waltham, MA) in a tungsten carbide vial containing zirconia ceramic balls (McMASTER-CARR, Robbinsville, NJ). The ball-milling process consisted of three 10-min sessions with a 5-min interval between consecutive sessions. After the ball milling process, 10 mL of chloroform (CAS#67-66-3, Fisher Scientific, Waltham, MA) was added to the grinding vial, and all materials, excluding the zirconia ceramic balls, were transferred to centrifuge tubes. The mixture was centrifuged at 750 rpm (66 rcf) for 10 min before the collection of the supernatant, which was centrifuged again at 8000 rpm (7500 rcf) for 10 min. The supernatant after the second centrifugation step was discarded, and the pellet was re-dispersed in chloroform with sonication until all pellets were re-dispersed in chloroform. The resulting solution was then transferred to a glass vial containing methoxy polyethylene glycol distearoylphosphatidylethanolamine (mPEG-DSPE) (5000 Da, Jenkem Technology, Plano, TX) with a weight ratio of 40 mg mPEG-DSPE per 1 g of Sr_4_Al_14_O_25_:Eu,Dy (based on the pre-ball-milling weight of the bulk material), and the mixture was sonicated to facilitate PEGylation. After PEGylation, chloroform was removed by blow-drying, leaving the PEGylated MLNTs in the vial. The dried PEGylated material was re-dispersed in 10 mL of deionized (D.I.) water with sonication. The resulting aqueous solution was centrifuged at 8000 rpm (7500 rcf) for 10 min, and the supernatant was discarded. The final pellet was re-dispersed in 1 mL of D.I. water, and then left to sit at 1 g for 40 min, after which the supernatant solution was collected for further experimental use.

### Structure and morphology characterizations.

Scanning electron microscopy (SEM) images of the bulk mechanoluminescent material were obtained using an Apreo S LoVac SEM (Thermo Fisher Scientific, Waltham, MA). Transmission electron microscopy (TEM) images of MLNTs were captured with a Tecnai TEM (FEI Company, Hillsboro, OR). X-ray diffraction (XRD) patterns of the bulk mechanoluminescent material and MLNTs were measured using a PANalytical Empyrean diffractometer (Malvern Panalytical Ltd., Malvern, United Kingdom).

### Evaluation of MLNT stability.

MLNTs were dispersed in D.I. water or 1× phosphate-buffered saline (PBS) in glass vials. A 20 μL aliquot of the solution was pipetted onto a glass slide. The brightfield image was first acquired, followed by charging the solution with a UV flashlight. Afterglow images were then acquired using a scientific complementary metal-oxide semiconductor (CMOS) camera (CS165MU, Thorlabs, Newton, NJ). To assess the MLNT solution stability in both conditions, aliquots were taken from the same vial every day, and the same imaging procedure was performed for 7 consecutive days using the same imaging parameters.

### Optical characterizations of the ultrasound-mediated light source.

The photoluminescence excitation and emission spectra of bulk mechanoluminescent materials and MLNTs were measured by a FluoroLog fluorometer (Horiba, Kyoto, Japan). For mechanoluminescence characterizations, an MLNT-doped polydimethylsiloxane (PDMS) phantom (30 mg/mL) was prepared as previously described^65^. The PDMS phantom was pre-charged with a 365-nm UV-LED (1 mW/mm^2^, SOLIS-365C, Thorlabs, Newton, NJ) before its mechanoluminescence emission was characterized. Transducers with a central frequency of 0.65 MHz, 1.5 MHz (Image Guided Therapy, Pessac, France), 3.5 MHz (Precision Acoustics, Dorchester, United Kingdom), and a homemade wearable transducer with a central frequency of 5.7 MHz (see “Fabrication and validation of the wearable transducer” for fabrication details below) were used. Specifically, for mechanoluminescence spectral measurements, mechanoluminescence spectrum was acquired by an OCEAN-HDX-VIS-NIR spectrometer (Ocean Insight, Orlando, FL) while FUS was applied by the 1.5-MHz ring transducer at a repetition rate of 1 Hz, and a duty cycle of 20%. Additionally, the mechanoluminescence emission in the phantom generated by FUS pulses at varied pressures (**Supplementary Fig. 2**) was also captured by the CMOS camera. Besides, for imaging the emission pattern under raster-scanning FUS, FUS was applied by the 5.7-MHz wearable transducer with a repetition rate of 1 Hz, and a duty cycle of 20% while the wearable transducer was repositioned to varied locations.

### Measurements of the FUS-induced mechanoluminescence emission in the artificial circulatory system.

The setup of the artificial circulatory system is illustrated in Fig. 3a & **Supplementary Fig. 4a,** and a detailed description can be found in our previously published work^65^. Briefly, the MLNT solution in D.I. water (9 mg/mL) filled a Tygon tubing (McMaster-Carr, Robbinsville, NJ) with the inner and outer diameters of 1.59 mm and 3.18 mm, respectively. The MLNT solution was circulated in the tubing by a peristaltic pump (Model 720, Harvard Apparatus, Holliston, MA) with a flow rate of 11.3 mL/min. The tubing was mounted in the center of the ring transducer with pre-filled ultrasound gel (Parker Aquasonic, Fairfield, NJ) by a 3D-printed holder. A 365 nm LED (M365LP1, Thorlabs, Newton, NJ) with a power density of 1 mW/mm^2^ was employed to recharge the solution before the solution was stimulated by the FUS pulses with a repetition rate of 1 Hz and a duty cycle of 20% generated by the ring transducer. The FUS-induced mechanoluminescence emissions from the MLNT solution were recorded by the CMOS camera. In addition, a photodiode (SM05PD1A, Thorlabs, Newton, NJ) was used to measure the absolute power of the mechanoluminescence emission with a distance of 4.5 mm between the inner surface of the tubing and the active area of the photodiode.

### Vertebrate animal subjects.

All animal procedures were approved by Stanford University’s Administrative Panel on Laboratory Animal Care (APLAC) and the University of Virginia Institutional Animal Care and Use Committee (IACUC). Mice had access to food and water ad libitum and were kept in facilities maintained for 12 h light/dark cycle. Adult C57BL/6J mice (male and female, 6–9 weeks old) were received from the Jackson Laboratory (000664) and allowed to acclimate for at least 3 days before enrolling them in experiments. Thy1-ChR2-YFP mice (Jackson Laboratory, 007612), D1-cre mice (a gift from Guler lab at the University of Virginia, Drd1^tm1(cre)Rpa^, MGI:3527153), and A2a-cre mice (Mutant Mouse Resource and Research Center (MMRRC), 036158) were housed and bred according to APLAC and IACUC guidelines. Mice bred in-house were used for experiments at 6–9 weeks of age. D1-cre mice and A2a-cre mice were genotyped to confirm the transgene expression before experimental use. Both sexes were used unless stated otherwise.

### Measurement of MLNTs concentration in blood after systemic administration.

C57BL/6J mice (n = 3) were anesthetized by 1–3.5% isoflurane and MLNTs in 1× PBS solution (200 μL of 30 mg/mL) were systemically delivered to the animals’ blood circulation via retro-orbital injection. Following the injection, an amount of 2.5 μL of blood was collected from the tail vein at multiple time points (30 s, 1 min, 3 min, 10 min, 30 min, and 60 min). Blood samples were then dissolved in 35% HNO_3_ at 70°C and were diluted 2,000 times by D.I. water and measured by inductively coupled plasma mass spectrometry (ICP-MS, Thermo Fisher Scientific, Waltham, MA). The percent injected dose per gram of blood sample (%ID/g) in each time point was obtained by normalizing the amount of MLNTs in the blood sample against both the initial injected dose and the blood sample mass. A first-order exponential decay was fitted to the data to estimate the circulation half-life of systemically administered MLNTs.

### Imaging of the mechanoluminescence emission from the mouse through the ring transducer.

C57BL/6J mice were anesthetized and humanely euthanized to allow for the dissection and removal of overlying tissues, enabling direct visualization of light emission from deep-seated organs (e.g., brain, gut, muscle, and spine). These mice were transcardially perfused with 10 mL of 1× PBS followed by 2.5 mL of MLNT solution at a concentration of 30 mg/mL. The scalp and skull were removed to facilitate imaging due to substantial scattering of blue mechanoluminescence through the scalp and skull. The mouse was subsequently mounted on a head holder (SG-4N, Narishige, Tokyo, Japan). The ring transducer with pre-filled ultrasound gel was then lowered to be positioned over the exposed mouse brain, while an electron-multiplying CCD (EMCCD; iXon Ultra 888, Andor Technology, Belfast, UK) was used for imaging the mechanoluminescence emission from the brain through the central opening of the ring transducer with simultaneous FUS stimulation with a repetition rate of 1 Hz and a duty cycle of 20%. Similarly, the skin on the mouse belly, limb, and back was removed to expose the gut, muscle, and spine, respectively. A CMOS camera was then used to capture mechanoluminescence emissions from these regions through the ring transducer under the same FUS stimulation conditions.

### *In vivo* electrophysiological recording.

A customized-made flexible fiber probe^37^ was used to avoid mechanical noise induced by the FUS pulses. Two electrodes were manually connected to the pin connector (Sullins Connector Solutions, San Marcos, CA) with copper wire and silver paint (SPI Supplies, West Chester, PA). Adult Thy1-ChR2-YFP mice were positioned in a stereotaxic apparatus (David Kopf Instruments, Los Angeles, CA) and 1–3.5% isoflurane was induced to animals via a nose cone. A heating pad (SomnoSuite, Kent Scientific, Torrington, CT) was used to maintain the mouse’s body temperature at 37 °C during anesthesia. Depilatory cream (Nair, Church & Dwight, Ewing, NJ) was used to remove the hair from the mouse head and back, and ophthalmic ointment was placed on both eyes. To expose the skull, a small incision was made in the scalp along the midline and the skull was further exposed by pulling the scalp to the sides. Following skull exposure, two small craniotomies were made with a dental drill, with one targeting the motor cortex for fiber probe implantation (1 mm anteroposterior (AP); −1.5 mm mediolateral (ML); −1 mm dorsoventral (DV) relative to bregma) and the other one targeting the cerebellum for stainless steel ground wire (AISI 316 alloy, Goodfellow, Pittsburgh, PA) implantation (−6 mm AP, −2 mm ML, and −2 mm DV relative to bregma). Before the probe implantation, the fiber probe was connected to a recording system consisting of an RHD 32-channel headstage and an RHD 2000 Intan 512ch Recording Controller (Intan Technologies LLC, Los Angeles, CA). The fiber probe was then placed through the hole of the ring transducer with pre-filled ultrasound gel, and the fiber probe and the ring transducer were separately mounted on the stereotaxic frame. Then the fiber probe was lowered using a micropositioner to the targeted coordinates for the motor cortex provided above. The ground wire was implanted in the cerebellum according to the coordinates provided above. Following the probe implantation, the ring transducer was lowered such that the focus of the ring transducer reached the same targeted brain region as the electrode. FUS pulses generated by the ring transducer were controlled by a 4-channel optogenetic controller, PlexBright (Plexon, Dallas, TX), and were recorded as digital signals by the Intan recording system. The sampling frequency of the electrophysiological recording was 20,000 Hz and FUS was applied with a repetition rate of 1 Hz or 10 Hz and a duty cycle of 20%. For animals receiving the MLNTs administration, the ring transducer was first raised by 10 cm via the stereotaxic manipulator, and 200 μL of MLNTs in 1× PBS solution (30 mg/mL) was retro-orbitally injected. After the injection, the ring transducer was lowered by 10 cm and 365-nm light (1 mW/mm^2^) from a UV-LED (SOLIS-365C, Thorlabs, Newton, NJ) was applied to the depilated area of the mouse back for recharging circulating MLNTs.

### Electrophysiological data analysis.

Data analysis was performed with MATLAB (Mathworks, Natick, MA) and custom scripts were used to sort neural spikes. The activities were digitally filtered in the range of 250–6000 Hz to present single spike resolutions. The spike sorting algorithm was implemented by filtering out individual spikes using a threshold determined by the standard deviation of the baseline, reducing the dimensionality of the data via principal component analysis (PCA), and using K-means clustering algorithms to separate the clusters.

### Ultrasound-mediated photostimulation on anesthetized Thy1-ChR2-YFP mice.

Adult Thy1-ChR2-YFP mice were used in this study and were anesthetized via intraperitoneal injection of a ketamine (80 mg/kg) and dexdomitor cocktail (1 mg/kg). To minimize baseline c-Fos activity in the targeted brain region, animals were maintained under anesthesia for 2 h on a heating pad (Harvard Apparatus, Holliston, MA) at 37 °C to prevent hypothermia before proceeding with the stimulation procedures. Depilatory cream was applied to remove the fur from the head and back, and a small incision was made on the scalp along the midline to expose the skull. Ophthalmic ointment was placed on both eyes. Subsequently, the head was mounted on a head holder and the ring transducer was positioned above the head with ultrasound gel applied. An “L” shaped aligner was used to promote the FUS targeting accuracy as previously described^65^. The coordinates relative to bregma used in this study are: primary motor cortex (M1): 1 mm AP, −1.5 mm ML, and −1 mm DV; primary somatosensory cortex (S1): −2 mm AP, −1.5 mm ML, and −1 mm DV; primary visual cortex (V1): −3.5 mm AP, −2 mm ML, and −1 mm DV; dorsal dentate gyrus (dDG): −2 mm AP, −1 mm ML, and −2 mm DV; and ventral dentate gyrus (vDG): −3 mm AP, −2.5 mm ML, and −2.5 mm DV.

Similar to the stimulation described in the electrophysiology section, the ring transducer was raised to facilitate the injection of MLNTs (200 μL of 30 mg/mL) and then lowered back to its designated location. FUS stimulation was applied with a repetition rate of 1 Hz and a duty cycle of 20% while 365-nm recharging light was applied to the back from a UV-LED with an incident power density of 1 mW/mm^2^. The FUS pulse train lasted 3 min if a single region was stimulated per animal, and 1 min in each targeted region if multiple regions were stimulated per animal. For single-region (M1 or vDG, coordinates as mentioned above) experiments, three control conditions were included: (1) the contralateral side (i.e., the corresponding brain region in the right hemisphere without FUS stimulation) from the same animal receiving both MLNTs and FUS; (2) another set of animals receiving vehicle (i.e., 1× PBS) injection with FUS in the left hemisphere; (3) a third set of animals receiving MLNTs injected and recharged in the back but without FUS. To validate region-specific c-Fos expression levels across multiple brain regions following single focal FUS application, three experimental groups were included, in which FUS was selectively applied to M1, S1, or V1, respectively. For stimulation in each of these brain regions, the other non-stimulated brain regions were used as controls.

Direct photostimulation of the M1 region through the intact skull using a light-emitting diode (LED) was included as an additional control, with the hypothesis that the conventional light sources cannot deliver sufficient optical power for effective activation of neurons even in shallow cortical regions without craniotomy. Specifically, a multimode fiber (M63L01, Thorlabs, Newton, NJ) coupled to a fiber-coupled LED (470 nm, M470F4, Thorlabs, Newton, NJ) was positioned directly against the skull surface. Following the fiber placement, direct photostimulation was delivered from the fiber tip at an output power density of 5 mW/mm^2^, targeting coordinates of 1 mm AP, −1.5 mm ML, with a frequency of 1 Hz and a 20% duty cycle, and a total duration of 3 min.

For multi-region stimulation, two experimental conditions were performed: (1) FUS pulses were delivered to M1, S1, and V1 on the left hemisphere of the same animal by adjusting the transducer position in the anteroposterior and mediolateral directions; (2) FUS pulses were delivered to M1 and dDG on the left hemisphere of the same animal by repositioning the transducer along the mediolateral, anteroposterior, and dorsoventral axes. The contralateral regions corresponding to the stimulated sites were used as controls. All animals were transcardially perfused 90 min after the procedures, first with 10 mL of 1× PBS, followed by 20 mL of 4% paraformaldehyde (PFA), and brains were collected and fixed in 4% PFA for 24 h.

### Fabrication and validation of the wearable transducer.

A 5.7-MHz lead zirconate titanate (PZT) ceramic disc (DL-48, Del-Piezo Specialties, West Palm Beach, FL) with an 8-mm diameter and a 10-mm focal distance was employed as the active element to generate sufficient acoustic energy. Silver electrodes were deposited on both the top and bottom surfaces of the PZT disc. A custom-fabricated brass housing featuring a lateral aperture was used, into which a SubMiniature version A (SMA) connector was integrated to interface with the bottom electrode of the PZT element. Subsequently, a degassed epoxy (EpoTek 301, Epoxy Technology, Inc., Billerica, MA) was dispensed into the brass housing to encapsulate and secure the connection between the SMA connector and the PZT disc after curing. Afterwards, a Cr/Au (50 nm/100 nm) electrode was sputtered onto the top surface of the PZT disc and the adjacent area of the brass housing to serve as a ground connection, using a commercial sputter coater (NSC-3000 Sputter Coater, Nano-Master, Inc., Austin, TX). Finally, a 15-μm thick layer of Parylene C (Specialty Coating Systems, Indianapolis, IN) was uniformly vapor-deposited over the entire external surface of the transducer using a parylene coater (PDS 2010, Specialty Coating Systems, Indianapolis, IN). Pulse-echo performance of the fabricated 5.7 MHz ultrasound transducer was measured by an ultrasonic pulser-receiver (Panametrics 5900PR, Olympus NDT Inc., Waltham, MA).

### Assembly of the head-mounting system.

The head-mounting system (**Supplementary Fig. 12**) consists of a headbar, a transducer holder, 2 nuts (M1.6), 2 screws (M1.6), and 2 mounting caps for securing the transducer onto the mouse head. The head-mounting system was designed to allow the wearable transducer to target the striatum regions of both hemispheres of the mouse brain (0 mm AP, ±1.5 mm ML, −3 mm DV). The transducer was inserted into the upper guiding channel of the transducer holder, which provided a tight fit that restricted its movement to a single mediolateral axis. Specifically, the repositioning of the wearable transducer from left to right hemisphere was easily achieved by adjusting the screws on both sides of the transducer holder.

### Neuron-type specific viral transduction.

pAAV-EF1a-double floxed-hChR2(H134R)-EYFP-WPRE-HGHpA was a gift from Karl Deisseroth (Addgene viral prep # 20298-PHPeB, http://n2t.net/addgene:20298; RRID:Addgene_20298, Addgene, Watertown, MA). Adult D1-Cre and A2a-Cre mice were anesthetized by 1–3.5% isoflurane, followed by retro-orbital injection of AAV-PHP.eB-EF1a-double floxed-hChR2(H134R)-EYFP-WPRE-HGHpA with a dose of 1.1–2.2 × 10^11^ vg/mouse to produce D1-Cre::ChR2-YFP and A2a-Cre::ChR2-YFP mice, or viral vehicle AAV-PHP.B-EF1a-double floxed-EYFP (Biohippo, Gaithersburg, MD) with a dose of 1–2 × 10^11^ vg/mouse to produce D1-Cre::YFP and A2a-Cre::YFP mice. Following injection, mice were allowed to recover and maintained for at least 4 weeks to ensure sufficient viral expression before subsequent procedures.

### Dynamic ultrasound-mediated photostimulation on anesthetized viral-transduced mice.

D1-Cre::ChR2-YFP mice, D1-Cre::YFP mice, A2a-Cre::ChR2-YFP mice, and A2a-Cre::YFP mice received surgery for a 3D-printed headbar attachment. Specifically, depilatory cream was applied to the scalp and back for hair depilation, followed by a midline incision of the scalp to expose a circular region of the skull with a diameter of approximately 6 mm. Ophthalmic ointment was placed on both eyes. Dental cement was applied to the headbar surface contacting the bone, with the center of the headbar aligned with bregma. The headbar was secured with additional cement, and the scalp was closed around it using skin glue. To protect the skull from direct exposure, silicone elastomer (Kwik-Sil, World Precision Instruments, Sarasota, FL) was applied as a sealing layer. Animals were allowed to recover for at least 7 days before dynamic ultrasound-mediated photostimulations. Before dynamic ultrasound-mediated photostimulation, the animals were anesthetized with ketamine/xylazine (60–80 mg/kg ketamine, 5–10 mg/kg xylazine) and were maintained under anesthesia for 2 h on a heating pad (SomnoSuite) at 37 °C to prevent hypothermia before proceeding with the stimulation procedures. The silicone elastomer was then removed, and the wearable transducer was subsequently mounted on the mouse head with ultrasound gel filled in the transducer holder using the head-mounting system. MLNTs in 1× PBS solution (200 μL of 30 mg/mL) were retro-orbitally injected, followed by FUS stimulation at a repetition rate of 1 Hz and a duty cycle of 20%. Stimulation was first applied to the aforementioned target (0 mm AP, −1.5 mm ML, −3 mm DV) in the left hemisphere for 3 min. The transducer was then repositioned to the targeted region (0 mm AP, 1.5 mm ML, −3 mm DV) on the right hemisphere, and an additional 3-min stimulation was performed. Back recharging was conducted using 365-nm UV light (1 mW/mm^2^) from a UV-LED. Three control groups were included for both D1-Cre and A2a-Cre mouse lines, each designed to exclude one key component (MLNTs, FUS, or ChR2 expression): (1) D1- and A2a-Cre::ChR2-YFP, FUS+ on the left hemisphere, MLNTs− (i.e., 1× PBS injection); (2) D1- and A2a-Cre::ChR2-YFP, FUS-, MLNT+; (3) D1- and A2a-Cre::YFP, FUS+ on the left hemisphere, MLNT+. 90 min after the procedures, all animals were transcardially perfused with 10 mL of 1× PBS, followed by 20 mL of 4% PFA, and brains were collected and soaked in 4% PFA for 24 h for further fixation.

### Behavioral Experiments.

C57BL/6J mice, D1-Cre::ChR2-YFP mice, D1-Cre::YFP mice, A2a-Cre::ChR2-YFP mice, and A2a-Cre::YFP mice were used in behavioral experiments and received headbar attachment as described above. The wearable transducer was connected to a commutator (SL2C, Plastics1, Roanoke, VA), which was positioned through a hole located in the center of the roof of a transparent cage. After recovering from headbar attachment (at least 7 days), each mouse was habituated in the transparent cage with the wearable transducer mounted on its head for 30 min per day for 2–3 consecutive days. Following habituation, animal movement was recorded using EthoVision XT 14 software (Noldus Information Technology, Leesburg, VA), with the camera placed underneath the cage, recording the mice from below. EthoVision software detected the coordinates of the nose, tail, and body center of each mouse. To evaluate the behavioral impact of tethering and transducer weight imbalance, one group of C57BL/6J mice was sequentially tested under four conditions, including three transducer placements (left, right, midline) and a control without tethering.

For dynamic ultrasound-mediated photostimulation on free-behaving D1-Cre::ChR2-YFP and A2a-Cre::ChR2-YFP mice, depilatory cream was applied to the back for hair removal 1 day before the ultrasound-mediated photostimulation. Each mouse was then anesthetized with 1–3.5% isoflurane, followed by retro-orbital injection of MLNTs in 1× PBS solution (200 μL of 30 mg/mL), the removal of the silicone elastomer, and the assembly of the head-mounting system on its head. The wearable transducer was initially placed on the left side of the holder. After recovery from anesthesia for 5 min in the cage, FUS stimulation with a repetition rate of 1 Hz and a duty cycle of 20% was applied for approximately 1 min with simultaneous back recharging. Following stimulation on the left side, the wearable transducer was repositioned to the right side, and the same FUS stimulation and back recharging were applied. Three control groups were included for both D1-Cre and A2a-Cre mouse lines, in which the FUS transducer was repositioned from the left to the right hemisphere regardless of stimulation condition. Each control group was designed to isolate the contribution of MLNTs, FUS stimulation, or ChR2 expression: (1) D1- and A2a-Cre::ChR2-YFP, FUS+, MLNT− (i.e., 1× PBS injection); (2) D1- and A2a-Cre::ChR2-YFP, FUS-, MLNT+ (with recharging); (3) D1- and A2a-Cre::YFP, FUS+, MLNT+.

### Mean square displacement (MSD) behavior analysis.

The MSD was calculated based on the animal’s body center coordinates and was computed as a function of time lag using the equation:

$$\text{MSD}(\tau )=\frac{1}{N-\tau }\sum_{i=1}^{N-\tau }\left[{\left(x_{i+\tau }-x_{i}\right)}^{2}+{\left(y_{i+\tau }-y_{i}\right)}^{2}\right]$$

where N is the total number of time points, and τ is the lag in frame number. The results were then converted into a time-resolved MSD by dividing τ by the frame rate (12.5 Hz) such that MSDs shown in **Supplementary Fig. 16** are expressed as a function of time in seconds.

### Rotational behavior analysis.

To quantify the rotational behavior of the animal, angular displacement was calculated based on the head-body vector, which represents the direction from the body center to the nose. At each frame, the orientation of the head-body vector was determined relative to a fixed axis, and angular displacement was calculated as the cumulative change in orientation over time, referenced to *t* = 0 s. Angular changes between consecutive frames were computed and normalized to the range [−π,π] to ensure continuity across angular boundaries. The cumulative angular displacement, expressed in revolutions, was obtained by summing the frame-to-frame angular changes and divided by 2π. Clockwise rotations were defined as negative angular displacements, while counter-clockwise rotations were defined as positive, while the behaving mice were imaged by a camera placed underneath the cage

### Biocompatibility assessment of the administered MLNTs and FUS pulses.

Four groups of C57BL/6J mice (n = 4 per group) were used to assess the effects of MLNTs and FUS stimulation on the brain. Two groups received an intravenous injection of MLNTs (200 μL, 30 mg/mL) and transcardially perfused at 1 week and 4 weeks, respectively, while the other two groups received 1× PBS and were perfused at the same time points as controls. FUS pulses with a repetition rate of 1 Hz and a duty cycle of 20% were applied to the left hemisphere of these mice at coordinates relative to bregma: 1 mm AP, −1.5 mm ML, and −1 mm DV following injection. The contralateral side (i.e., the right hemisphere) of each mouse brain was employed as the internal control without FUS stimulation. Mice were transcardially perfused with 10 mL of 1× PBS, followed by 20 mL of 4% PFA at either 1 week or 4 weeks post-injection, and brains were soaked in 4% PFA for 24 h for further fixation.

### Immunohistochemical (IHC) staining.

The brains collected in different experimental conditions were fixed in 4% PFA at 4 °C and 40-μm-thick coronal brain sections were sliced either on a cryostat (Leica CM 3050S, Leica Biosystems) or a vibratome (PELCO easiSlicer, Ted Pella, Redding, CA). When the cryostat was used, the fixed brain tissue was dehydrated in 30% sucrose solution at 4 °C before sectioning. After tissue sectioning, brain slices were washed three times in 1× PBS for 10 min each and then blocked at room temperature for 1 h in 1× PBS solution with 0.3% Triton X-100 and 5% goat/donkey serum (matched to the secondary antibody species; Jackson ImmunoResearch Laboratories). After blocking, slices were incubated in primary antibody solutions (Supplementary Table 1) in 1× PBS with 0.3% Triton X-100 and 5% goat/donkey serum overnight at 4°C. Following primary antibody incubation, slices were rinsed three times in 1× PBS with 0.05% Triton X-100 for 10 min each. Secondary antibodies (Supplementary Table 1), diluted with 0.1% Triton X-100 and 5% goat/donkey serum, were then applied for 1.5 h at room temperature. After three final washes in 1× PBS for 10 min each, slices were mounted onto glass slides and coverslips with mounting media ProLong Gold Antifade Mountant (Invitrogen). Confocal fluorescence images were captured using a Zeiss LSM 780 and an Olympus FV3000 confocal microscope.

### Brain temperature monitoring under FUS application

Similar to the procedures described in the “*In vivo* electrophysiological recording” section, a K-Type thermocouple probe (DigiKey, Thief River Falls, MN) was implanted into the M1 region of a wild-type mouse brain following skull exposure and craniotomy. Brain temperature was recorded at 1 Hz while FUS (central frequency of 1.5 MHz, pressure of 2.3 MPa) was applied at the same repetition rate with a 20% duty cycle.

### *In vivo* biodistribution study.

Two groups of C57BL/6J mice (n = 4 per group) were retro-orbitally injected with 200 μL of MLNTs (30 mg/mL) in 1× PBS solution. The mice were transcardially perfused with 10 mL of 1× PBS at two time points of 1 day (24 h) and 7 days (168 h) post-injection. Major organs, including the heart, liver, spleen, lungs, kidneys, and brain, were collected, weighed, and dissolved in 35% HNO_3_ at 70°C. The samples were then diluted 100-fold with D.I. water and analyzed using ICP-MS. The percentage of the injected dose per gram of tissue (%ID/g) was calculated by normalizing the detected MLNT amount to both the initial injection dose and the tissue mass.

### H&E staining for tissue damage evaluation.

Four groups of C57BL/6J mice (n = 4 per group) were used to evaluate the long-term effects of systemically administered MLNTs and the vehicle of 1 × PBS solution. Two groups received retro-orbital injection of 200 μL of MLNTs (30 mg/mL) or 1× PBS and were transcardially perfused with 10 mL of 1× PBS followed by 20 mL of 4% PFA after 1 week. The remaining two groups received the same injections and were transcardially perfused the same way after 4 weeks. Major organs, including the brain, heart, liver, spleen, lungs, and kidneys, were collected and fixed in 4% PFA for 24 h. After fixation, the tissues were embedded in paraffin, sectioned into 5-μm slices, and stained with hematoxylin and eosin (H&E). The stained sections were imaged using a Keyence BZ-X810 microscope (Keyence, Itasca, IL).

### Metabolic study for quantifying excretion of injected MLNTs.

Two groups of C57BL/6J mice (n = 4 per group) were individually housed in metabolic cages after retro-orbital injection of 200 μL of MLNTs (30 mg/mL) in 1× PBS solution. Feces and urine samples were collected over 7 days. The samples were weighed, dissolved in 35% HNO_3_ at 70°C, and then diluted 100 times with D.I. water. The MLNTs were quantified using ICP-MS, and the percentage of the injected dose per gram of sample (%ID/g) was calculated by normalizing the detected MLNT amount to both the initial injection dose and the sample mass.

### Replication

The sample size for each experiment was determined by power analysis to ensure statistical rigour for all comparisons^66^. Mechanoluminescence imaging from mice systemically administered with MLNTs was repeated in 4 mice. Electrophysiological recording was repeated in 5 mice. Single-region stimulation was repeated in 3 mice per experimental condition, resulting in 21 mice. Cross-validating c-Fos expression in single brain regions was repeated in 3 mice per experimental condition, resulting in 9 mice. Multi-region stimulation was repeated in 3 mice per experimental condition, resulting in 6 mice. Evaluation of tethering on animal behavior was repeated in 3 mice. Dynamic neuron-type specific neuromodulation on anesthetized animals was repeated in 3 mice per experimental condition, resulting in 24 mice. Dynamic neuron-type specific neuromodulation in free-behaving animals was repeated on 5 mice per experimental condition, resulting in 40 trials. Biodistribution of systemically delivered MLNTs was repeated in 4 mice per experimental condition, resulting in 8 mice. Measurement of MLNTs concentration in the bloodstream was repeated in 3 mice. The assessment of chronic immune response was repeated in 4 mice per experimental condition, resulting in 16 mice. The *in vivo* clearance study of intravenously administered MLNTs was repeated in 4 mice. Complete blood count (CBC) tests were repeated in 4 mice per experimental condition, resulting in 16 mice. Histopathological analysis of mouse organs was repeated in 4 mice per experimental condition, resulting in 16 mice.

### Statistical analysis

Comparison between groups was evaluated using one-way analysis of variance (ANOVA) in OriginPro software, with P < 0.05 considered statistically significant.

## Supplementary Material

Supplementary Files

This is a list of supplementary files associated with this preprint. Click to download.

- SupplementaryInformation.pdf
- SupplementaryMovie1.avi
- SupplementaryMovie2.avi
- SupplementaryMovie3.avi
- SupplementaryMovie4.avi
